# Integrated microbiome and metaproteome analyses reveal microbial functions underlying traditional fermented amaranth stem fermentation

**DOI:** 10.3389/fmicb.2026.1983810

**Published:** 2026-10-30

**Authors:** Lifei Meng, Hong Ze, Xilin Hu, Jian Chen, Kaimin Li, Qiqiong Li, Wentao Hu

**Affiliations:** 1 Department of Thoracic Surgery, The First Affiliated Hospital of Ningbo University, Ningbo, China; 2 State Key Laboratory of Food Science and Resources, Nanchang University, Nanchang, China

**Keywords:** correlation analysis, fermented amaranth stems, flavor, metaproteomics, microbial community

## Abstract

Fermented amaranth stems are a traditional Chinese fermented vegetable characterized by a distinctive pungent flavor and complex microbial ecology. This study compared samples from Anhui (AH), Jiangsu (JS), and Zhejiang (ZJ) using physicochemical analysis, volatile profiling, free amino acid (FAA) determination, 16S rRNA sequencing, and metaproteomics. Across the three regions, pH ranged from 4.08 to 4.39, total titratable acidity from 2.7 to 3.5 mL, viable counts from 7.7 to 8.6 log CFU/g, and total volatile concentrations from 99,021 to 332,864 μg/L. After removing chloroplast- and mitochondrial-derived reads, Firmicutes (approximately 56–77%) and Proteobacteria (approximately 23–40%) dominated the bacterial communities, with Lactobacillus, Lactococcus, and Pseudomonas as the major genera. Total FAA concentrations were 888.5, 778.0, and 582.0 μg/mL in AH, JS, and ZJ, respectively. Metaproteomics identified 7,158 unique protein accession records, of which 3,926 received taxonomic assignments. Functional annotation highlighted carbohydrate metabolism, energy metabolism, stress-response functions, and carbohydrate-active enzymes. These results demonstrate region-associated differences in bacterial composition, expressed functions, and flavor-related traits and provide candidate microbial–functional links for future targeted validation.

## 1 Introduction

Fermented vegetables are an important class of traditional foods worldwide because fermentation can improve sensory properties, extend shelf life, and reshape nutritional and bioactive components (Xu et al., 2024; Thierry et al., 2023). In spontaneous vegetable fermentation, indigenous microorganisms originating from raw materials, water, utensils, and processing environments form complex microbial ecosystems. Lactic acid bacteria, including Lactobacillus, Leuconostoc, Weissella, and Pediococcus, are frequently dominant, while non-LAB bacteria and yeasts may also contribute to substrate degradation, organic-acid metabolism, aroma formation, and ecological adaptation (Wang et al., 2026; Rosca et al., 2023). Consequently, raw-material microbiota, salt concentration, temperature, humidity, fermentation duration, and local processing practices can produce substantial differences in microbial community composition and metabolite profiles among fermented vegetable products (Xu et al., 2024; Thierry et al., 2023). Recent multi-omics studies have therefore moved beyond taxonomic description toward linking microbial composition with expressed functions and quality-related metabolites.

Fermented amaranth stems are a traditional vegetable product consumed particularly in the Jiangsu–Zhejiang region of China (Wang et al., 2025). Their spontaneous fermentation generates a distinctive pungent aroma and complex taste while transforming plant-derived carbohydrates and nitrogenous compounds into organic acids, free amino acids, and volatile metabolites. Previous studies on stinky fermented foods have identified indoles, organic acids, alcohols, ketones, phenolic compounds, and sulfur-containing compounds as important contributors to characteristic odor profiles, with indole being particularly relevant to fermented amaranth stems (Wang et al., 2025; Peng et al., 2025). More specifically, Peng et al. (2025) investigated the fermentation process of amaranth stems using metagenomics, flavoromics, and metabolomics and reported pronounced temporal changes in microbial composition and metabolites, including benzaldehyde, 2-nonanone, citronellol, isovaleric acid, and other flavor-related compounds. Pseudomonas, Acinetobacter, Pectobacterium, and Enterobacter were proposed as important functional microorganisms (Yan et al., 2025). These findings demonstrate that fermented amaranth stems harbor a more complex ecosystem than a simple LAB-dominated fermentation and that microbial metabolism is closely related to the transformation of nutritional and flavor-related components (Valentino et al., 2024).

Despite these advances, current studies on fermented amaranth stems have mainly focused on temporal changes within individual fermentation systems. Comparative information across production regions remains scarce, and little is known about whether similar products from different regions differ in their expressed microbial functions. Metagenomics can reveal community structure and functional genetic potential, but the presence of a gene does not necessarily indicate that the corresponding protein is expressed under a given fermentation condition (Wen et al., 2024). Metaproteomics therefore provides a complementary approach by directly characterizing proteins expressed by a mixed microbial community, allowing active metabolic pathways, enzyme systems, stress responses, and substrate interactions to be investigated (Bahule et al., 2024; Okeke et al., 2021; de Paula de Sousa Cavalcante et al., 2025). Integrating 16S rRNA profiling with metaproteomics can thus help bridge the gap between “which microorganisms are present” and “which functions are being expressed” in traditional fermentation systems (Vermeersch et al., 2026).

Accordingly, this study compared traditional fermented amaranth stems collected from Anhui, Jiangsu, and Zhejiang, China. Physicochemical properties, nitrite, viable counts, volatile compounds, and FAAs were characterized to define quality-related differences among the sampled products. Bacterial communities were evaluated using 16S rRNA gene sequencing, while metaproteomics combined with GO, KEGG, COG, and CAZy annotations was used to characterize expressed functional proteins. Correlation networks were then used to explore potential associations between dominant bacterial genera and functional categories. By integrating quality traits, bacterial community profiles, and expressed functions, this study aimed to identify region-associated patterns and generate testable hypotheses linking microbial composition, substrate transformation, acidification, and flavor formation. The findings provide a reference for future controlled fermentation, quality evaluation, and targeted mechanistic validation of this traditional fermented vegetable.

## 2 Materials and methods

### 2.1 Sample collection

Traditional fermented amaranth stem samples were obtained from three production regions in China: Guangde City (AH), Suzhou City (JS), and Ningbo City (ZJ). For each region, three independent samples were analyzed as biological replicates (*n* = 3). Each sample was homogenized and divided into subsamples for physicochemical characterization, viable cell enumeration, volatile flavor analysis, FAA determination, 16S rRNA gene sequencing, and metaproteomic analysis. Samples were immediately frozen in liquid nitrogen and stored at −80 °C until analysis. Because the products were obtained from spontaneous fermentations rather than a controlled common-process experiment, processing variables such as salt concentration, fermentation duration, temperature, production batch, manufacturer, and local environmental conditions could not be standardized. Therefore, the differences observed among AH, JS, and ZJ are interpreted as region-associated patterns rather than direct causal effects of geographical origin.

### 2.2 Determination of physicochemical and microbiological characteristics

The pH of fermented amaranth stems was measured using a calibrated digital pH meter according to GB 5009.237–2016. Total titratable acidity (TTA) was determined according to AOAC 942.15. Nitrite content was determined according to GB 5009.33–2016 while amino nitrogen was measured by formaldehyde titration according to GB 5009.235–2016. Amino nitrogen was expressed as g/kg.

For viable cell enumeration, samples were aseptically homogenized with sterile physiological saline, serially diluted tenfold, and plated on plate count agar (PCA) (Wang et al., 2023). Plates were incubated at 35 °C for 48 h. Plates within the countable range were selected, and results were expressed as log CFU/g.

### 2.3 Detection of volatile flavor compounds

Volatile organic compounds (VOCs) were analyzed by headspace solid-phase microextraction coupled with gas chromatography–mass spectrometry (HS-SPME–GC–MS) using an Agilent 8,890 GC coupled to a 5977C mass selective detector (Agilent Technologies, Santa Clara, CA, United States), following a previously reported method with minor modifications (Wang et al., 2024). Homogenized samples were centrifuged at 8,000 × g for 5 min at 4 °C. An aliquot (1.5 mL) of the supernatant and 6 μL of 2-octanol (4 mg/L) as the internal standard were transferred to a headspace vial. Samples were equilibrated at 60 °C for 10 min and extracted using a 30-μm DVB fiber at 50 °C for 30 min. The fiber was then desorbed in the GC injector at 250 °C for 5 min.

Separation was performed on a DB-5 capillary column (123–5,032, Agilent Technologies, Santa Clara, CA, United States) with helium at 1.0 mL/min. The oven program was 30 °C for 2 min, increased to 90 °C at 5 °C/min and held for 2 min, increased to 200 °C at 5 °C/min, and finally increased to 220 °C at 5 °C/min and held for 4 min. Mass spectra were acquired in electron-ionization mode at 70 eV, with an ion-source temperature of 250 °C, transfer-line/interface temperature of 280 °C, and scan range of m/z 30-500. Compounds were identified by comparison with the NIST mass spectral library. Quantification was performed using internal-standard semi-quantification.

### 2.4 Free amino acid analysis

The FAA profile was determined following the procedure reported previously (Zhong et al., 2021). Proteins were removed from the sample extracts to minimize interference from protein-associated amino acids. The extract and 10% sulfosalicylic acid were combined at a volume ratio of 4:1 and kept at 4 °C for 60 min. The mixture was centrifuged at 14,000 × g for 15 min using a, and the supernatant was filtered through a 0.22-μm membrane. FAA analysis was performed using an L-8900 automatic amino acid analyzer (Hitachi High-Technologies Corp., Tokyo, Japan) with external amino acid standards (79248-5X2ML, Sigma-Aldrich). Concentrations were reported as μg/mL of filtered extract. Because formaldehyde-titrated amino nitrogen represents a broader pool of titratable low-molecular-weight nitrogenous compounds than chromatographically quantified individual FAAs, the two indices were treated as complementary rather than directly equivalent measurements.

### 2.5 High-throughput sequencing

The bacterial community was characterized by high-throughput 16S rRNA gene sequencing according to the method reported previously (Meng et al., 2025). Genomic DNA was extracted using a CTAB–SDS protocol combined with liquid-nitrogen freeze–thaw treatment, and DNA quality was assessed by 1% agarose gel electrophoresis using a DYY-6C electrophoresis system (Beijing Liuyi Biotechnology Co., Ltd., Beijing, China). DNA concentration was determined using a NanoDrop 2000 UV–Vis spectrophotometer (Thermo Scientific, Wilmington, DE, United States) and normalized to 1 ng/μL. The V4 region was amplified using primers 515F (5′-GTGCCAGCMGCCGCGGTAA-3′) and 806R (5′-GGACTACHVGGGTWTCTAAT-3′). Amplicons were sequenced by Majorbio Bio-Pharm Technology Co., Ltd. (Shanghai, China) using an Illumina MiSeq PE300 platform (Illumina, San Diego, CA, United States), generating >30,000 clean reads per sample. Sequences were processed using fastp v0.20.0, FLASH v1.2.11, and UPARSE v7.1, and taxonomically assigned using RDP Classifier v2.2 against the SILVA 138 database. Before downstream bacterial analyses, chloroplast- and mitochondrial-derived sequences were removed and the remaining bacterial abundance table was renormalized. Alpha-diversity indices, phylum- and genus-level relative abundances, and PCA were recalculated from the filtered dataset. Because short V4 amplicons did not provide sufficiently robust species-level resolution, species-level comparisons and PCA were not interpreted.

### 2.6 Metaproteomic analysis

#### 2.6.1 Metaproteomic protein extraction

Proteins from fermented amaranth stem samples were isolated using a modified phenol-based extraction procedure (Bahule et al., 2024). Frozen samples were placed in grinding tubes, combined with an appropriate amount of BPP extraction buffer, and homogenized three times for 180 s using a FastPrep-96 tissue grinder (MP Biomedicals, Irvine, CA, United States) under frozen conditions. The homogenate was centrifuged at 14,000 × g for 15 min at 4 °C using a 5,430 R refrigerated centrifuge (Eppendorf, Hamburg, Germany). The supernatant was mixed with an equal volume of Tris-saturated phenol, vortexed for 5–10 s, and centrifuged under the same conditions. The phenolic phase was recovered, mixed with an equal volume of BPP buffer, and centrifuged again. Proteins were precipitated with four volumes of pre-cooled ammonium acetate/methanol overnight at −20 °C. The precipitate was collected by centrifugation and washed twice with 90% pre-cooled acetone. The pellet was dissolved in lysis buffer containing 8 M urea, 1% SDS, and a protease inhibitor cocktail and sonicated for 30 min using an SBL-22DT ultrasonic cleaner (Ningbo Scientz Biotechnology Co., Ltd., Ningbo, China). After centrifugation at 14,000 × g for 15 min at 4 °C, the supernatant was collected. Protein concentration was determined by BCA assay, and protein integrity was evaluated by SDS–PAGE before metaproteomic analysis.

#### 2.6.2 LC–MS/MS and protein identification

Peptide samples were analyzed using an UltiMate 3,000 liquid chromatography system (Thermo Fisher Scientific, Waltham, MA, United States) coupled to an Orbitrap Exploris 480 mass spectrometer (Thermo Fisher Scientific, Bremen, Germany) operated in data-independent acquisition (DIA) mode. Chromatographic separation was performed on a reversed-phase C18 analytical column using a formic-acid-containing water/acetonitrile gradient.

The acquired raw MS data were processed with Proteome Discoverer and searched against the MetaproteomicsDB database. Trypsin was specified as the digestion enzyme with up to two missed cleavages; cysteine carbamidomethylation was set as a fixed modification, whereas methionine oxidation and protein N-terminal acetylation were treated as variable modifications. Peptide/PSM and protein FDRs were controlled at ≤1%. Protein abundances were normalized by total peptide amount. The complete protein identification, abundance, functional annotation, and taxonomic assignment table is provided as Supplementary Table S1. Taxonomic assignments were recorded from domain to species level. For taxon-specific interpretation and microbiome–metaproteome integration, only proteins with microbial taxonomic assignments were considered; proteins without confident taxonomic assignments were not assigned to specific microorganisms.

### 2.7 Functional annotation of the metaproteome

Identified proteins were functionally annotated against four complementary databases (Wen et al., 2024). Gene Ontology (GO) annotation was used to classify proteins into biological process, cellular component, and molecular function categories. KEGG annotation was employed to characterize metabolic pathways and cellular functional modules. COG/eggNOG annotations were used to assign proteins to orthologous functional categories, whereas carbohydrate-active enzymes were classified according to the CAZy database.

### 2.8 Integrated 16S rRNA sequencing–metaproteomic correlation analysis

To investigate potential relationships between dominant bacterial taxa and expressed functional proteins, bacterial relative-abundance data obtained from the filtered 16S rRNA dataset were integrated with metaproteomic functional profiles (Wang et al., 2024). Dominant bacterial genera were selected based on relative abundance >1%, including Lactococcus, Lactobacillus, Pseudomonas, Leuconostoc, Chryseobacterium, Massilia, Flavobacterium, Delftia, and Rheinheimera where applicable. Only genera obtained from the actual genus-level abundance matrix were included. Normalized protein abundances assigned to GO terms, KEGG pathways, COG orthologs, and CAZy families were matched to the corresponding nine individual biological samples (*n* = 9), rather than to three regional means. Spearman’s rank correlation coefficients were calculated across samples, and associations satisfying |r| > 0.6 and *p* < 0.05 were retained as exploratory associations for network visualization (Yu et al., 2023).

Correlation networks were visualized using Cytoscape (3.10.4). Node size was proportional to the relative abundance of the corresponding bacterial genus or functional feature. Red edges represented positive correlations and green edges represented negative correlations; edge thickness was proportional to the absolute correlation coefficient.

### 2.9 Statistical analysis

All experiments were independently performed in triplicate, and quantitative results are reported as mean ± standard deviation (SD). Normality and homogeneity of variance were evaluated before parametric testing. Differences among AH, JS, and ZJ were assessed by one-way analysis of variance (ANOVA), followed by Duncan’s multiple-range test, with *p* < 0.05 considered statistically significant. Exact *p* values are reported in the revised text or Supplementary Table S1 where available. Multivariate microbial-community differences were evaluated by Bray–Curtis distance-based PERMANOVA with 999 permutations. Spearman’s rank correlations were calculated across the nine individual biological samples for exploratory microbiome–metaproteome integration; because of the limited sample size, these correlations were interpreted as hypothesis-generating rather than causal evidence. Statistical analyses were performed using SPSS Statistics 30 (IBM Corp., Armonk, NY, United States), and network visualization was performed using Cytoscape 3.10.4.

## 3 Results and discussion

### 3.1 The physicochemical and microbiological characteristics of traditional fermented amaranth stems

The three regional products exhibited distinct physicochemical and microbiological profiles, although all showed the acidic characteristics of vegetable fermentation. As shown in Figure 1A, JS had the highest pH (4.39 ± 0.06) and the lowest TTA (2.70 ± 0.08 mL), whereas AH showed the lowest pH (4.08 ± 0.04) and the highest TTA (3.50 ± 0.05 mL). This inverse relationship likely reflects differences in organic-acid accumulation and buffering capacity during spontaneous fermentation (Liang et al., 2022). Viable cell counts ranged from 7.7 ± 0.09 to 8.6 ± 0.16 log CFU/g (Figure 1B), with AH significantly higher than JS and ZJ (*p* < 0.05). However, viable counts alone cannot distinguish LAB from other microorganisms, which is relevant because fermented amaranth stems can harbor a more complex consortium than conventional LAB-dominated fermented vegetables (Peng et al., 2025).

**Figure 1:**
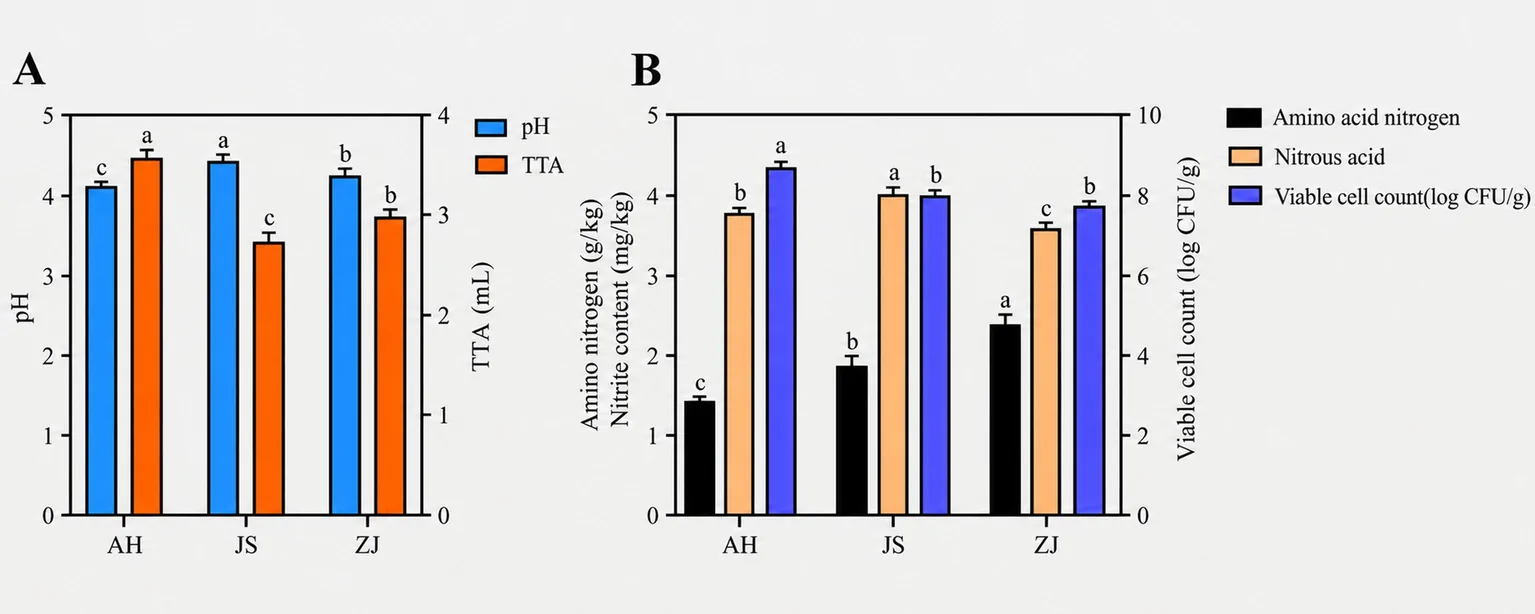
Regional variations in the physicochemical and microbiological characteristics of traditional fermented amaranth stems collected from Anhui (AH), Jiangsu (JS), and Zhejiang (ZJ), China. **(A)** pH and total titratable acidity (TTA); **(B)** amino nitrogen content, nitrite content, and viable cell count. Data are expressed as mean ± standard deviation (SD). Different lowercase letters above the bars indicate significant differences among regions for the same parameter (*p* < 0.05).

Amino nitrogen increased from AH to JS and ZJ (approximately 1.40 ± 0.05, 1.80 ± 0.07, and 2.35 ± 0.06 g/kg, respectively; *p* < 0.05), indicating greater accumulation of titratable soluble nitrogenous compounds in ZJ. Nitrite concentrations were 3.80 ± 0.05, 4.00 ± 0.04, and 3.60 ± 0.05 mg/kg in AH, JS, and ZJ, respectively. All values were below the maximum nitrite limit of 20 mg/kg (as NaNO₂) specified for pickled vegetables in GB 2762-2022. Nitrate was not measured. Because amino nitrogen and chromatographically quantified FAAs represent analytically different nitrogen pools, their regional trends were not assumed to be directly interchangeable.

### 3.2 Regional variation in volatile flavor profiles

The volatile profiles differed markedly among the three regional products. AH exhibited the highest total volatile concentration (332,864 μg/L), approximately 2.29- and 3.36-fold those of JS (145,109 μg/L) and ZJ (99,021 μg/L), respectively, and contained more detected compounds (45 vs. 39 in both JS and ZJ) (Figure 2A). Beyond total abundance, the differential compounds likely contributed differently to aroma quality. ZJ showed a greater relative contribution of acetic acid, which may reinforce acidic and pungent notes, whereas JS was enriched in 2-octanone, a ketone commonly associated with lipid- and microbial-derived aroma formation. Regional differences were also observed for 2,3-butanediol isomers, benzaldehyde derivatives, 2-nonanone, methyl salicylate, eugenol, and sulfur- and nitrogen-containing compounds (Figure 2B). Alcohols can act as both odorants and precursors of aldehydes, acids, and esters, while ketones may arise from lipid oxidation and microbial metabolism (Sun et al., 2023; Liang et al., 2020; Chen et al., 2021). Benzaldehyde-related compounds, methyl salicylate, and eugenol may contribute aromatic, sweet, spicy, or plant-like notes, whereas sulfur- and nitrogen-containing compounds can exert strong sensory effects even at low concentrations because of their low odor thresholds (Wang et al., 2025; Peng et al., 2025). Therefore, the regional flavor differences likely depend on both concentration and odor potency rather than total volatile abundance alone. Because odor activity values (OAVs) or GC–O data were not available for all compounds, these sensory assignments are considered literature-supported interpretations rather than direct sensory attribution.

**Figure 2:**
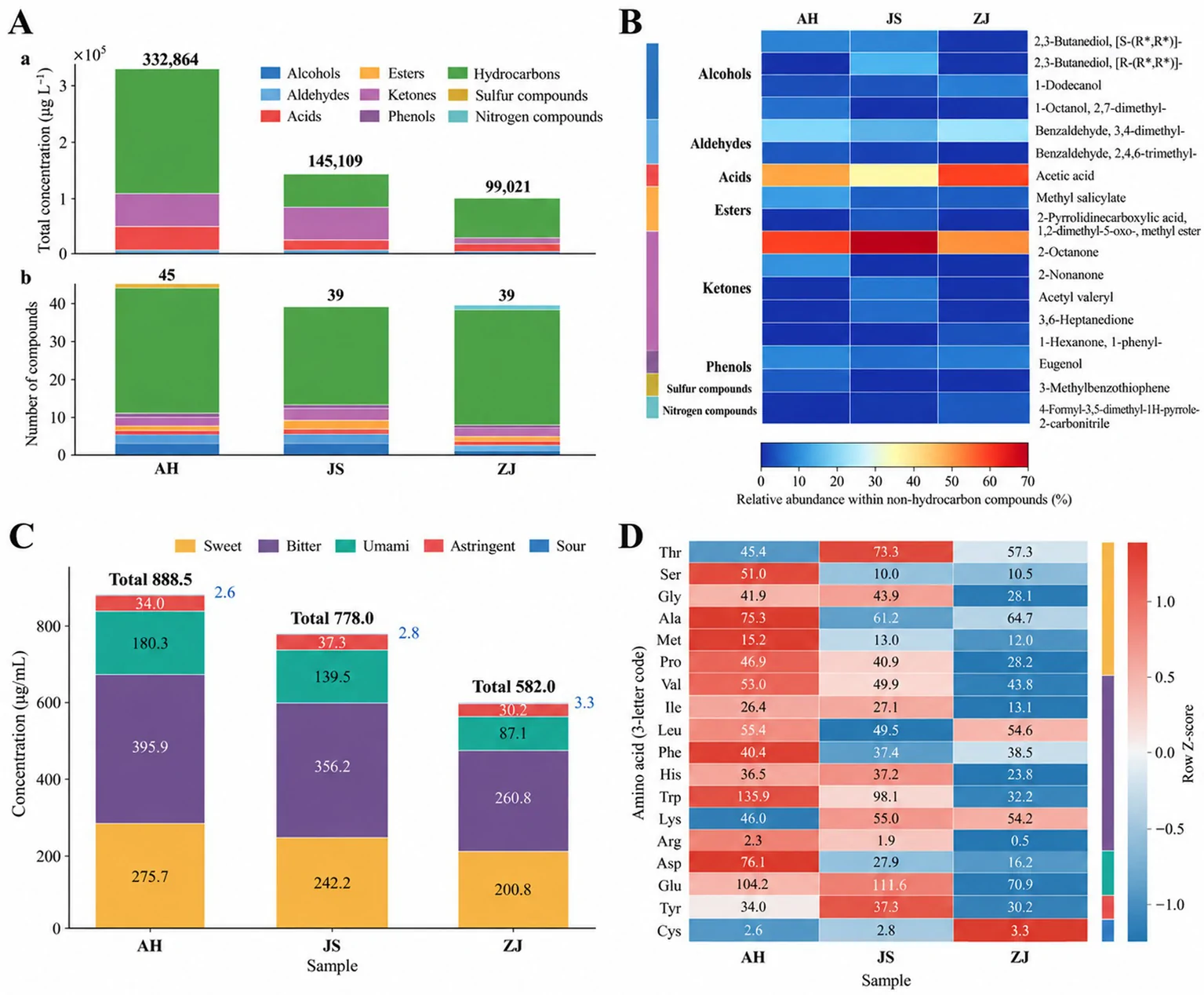
Regional differences in volatile flavor compounds and free amino acids (FAAs) of traditional fermented amaranth stems. **(A)** Total concentrations (upper panel) and numbers (lower panel) of volatile compounds classified as alcohols, aldehydes, acids, esters, ketones, phenols, hydrocarbons, sulfur compounds, and nitrogen-containing compounds. **(B)** Heatmap showing the relative abundance of representative non-hydrocarbon volatile compounds among samples. **(C)** Concentrations of FAAs grouped according to their major taste attributes, including sweet, bitter, umami, astringent, and sour tastes. Numbers above the bars indicate total FAA concentrations. **(D)** Heatmap of individual FAA concentrations. Values within cells represent FAA concentrations (μg/mL), whereas the color scale represents the row-wise Z-score. AH, Anhui; JS, Jiangsu; ZJ, Zhejiang. Different lowercase letters indicate significant differences among regions (*p* < 0.05), where shown.

### 3.3 The free amino acid composition and taste characteristics of fermented amaranth stem

Total FAA concentration decreased in the order AH (888.5 μg/mL) > JS (778.0 μg/mL) > ZJ (582.0 μg/mL) (Figure 2C). Bitter-taste amino acids represented the largest FAA fraction, reaching 395.9, 356.2, and 260.8 μg/mL in AH, JS, and ZJ, respectively, followed by sweet and umami FAAs. AH contained the highest total umami FAA level (180.3 μg/mL), while JS showed the highest individual Glu concentration (111.6 μg/mL). Trp showed the clearest regional difference, decreasing from 135.9 μg/mL in AH to 98.1 μg/mL in JS and 32.2 μg/mL in ZJ. Notably, the regional ranking of total FAA concentration was opposite to that of amino nitrogen. This does not necessarily indicate analytical inconsistency because formaldehyde titration measures a broader pool of titratable amino nitrogen, whereas the amino acid analyzer quantifies individual free amino acids after deproteinization. The two indices were therefore interpreted as complementary measurements of nitrogen transformation rather than as directly equivalent quantities. Sample-level correlation should be reported once the paired raw data and moisture/dry-matter information are verified. Trp utilization may contribute to indole formation, whereas Phe can serve as a precursor for phenylethanol biosynthesis (Peng et al., 2025).

### 3.4 Regional differences in microbial α-diversity

Following removal of chloroplast- and mitochondrial-derived sequences, α-diversity indices were recalculated using the filtered bacterial abundance table. Sobs, Shannon, Simpson, and Pielou indices were 1040.9 ± 6.6, 4.487 ± 0.018, 0.0425 ± 0.0014, and 0.6457 ± 0.0021 in AH; 1063.3 ± 1.7, 4.789 ± 0.006, 0.0293 ± 0.0003, and 0.6872 ± 0.0007 in JS; and 1065.2 ± 2.7, 4.798 ± 0.006, 0.0295 ± 0.0003, and 0.6883 ± 0.0011 in ZJ, respectively. AH differed significantly from JS and ZJ in Sobs (*p* < 0.01) and the other indices (*p* < 0.001), whereas JS and ZJ showed no significant differences. Only the post-filtering diversity indices are reported in Figure 3 and the main text to represent the bacterial community rather than plant organelle-derived sequences.

**Figure 3:**
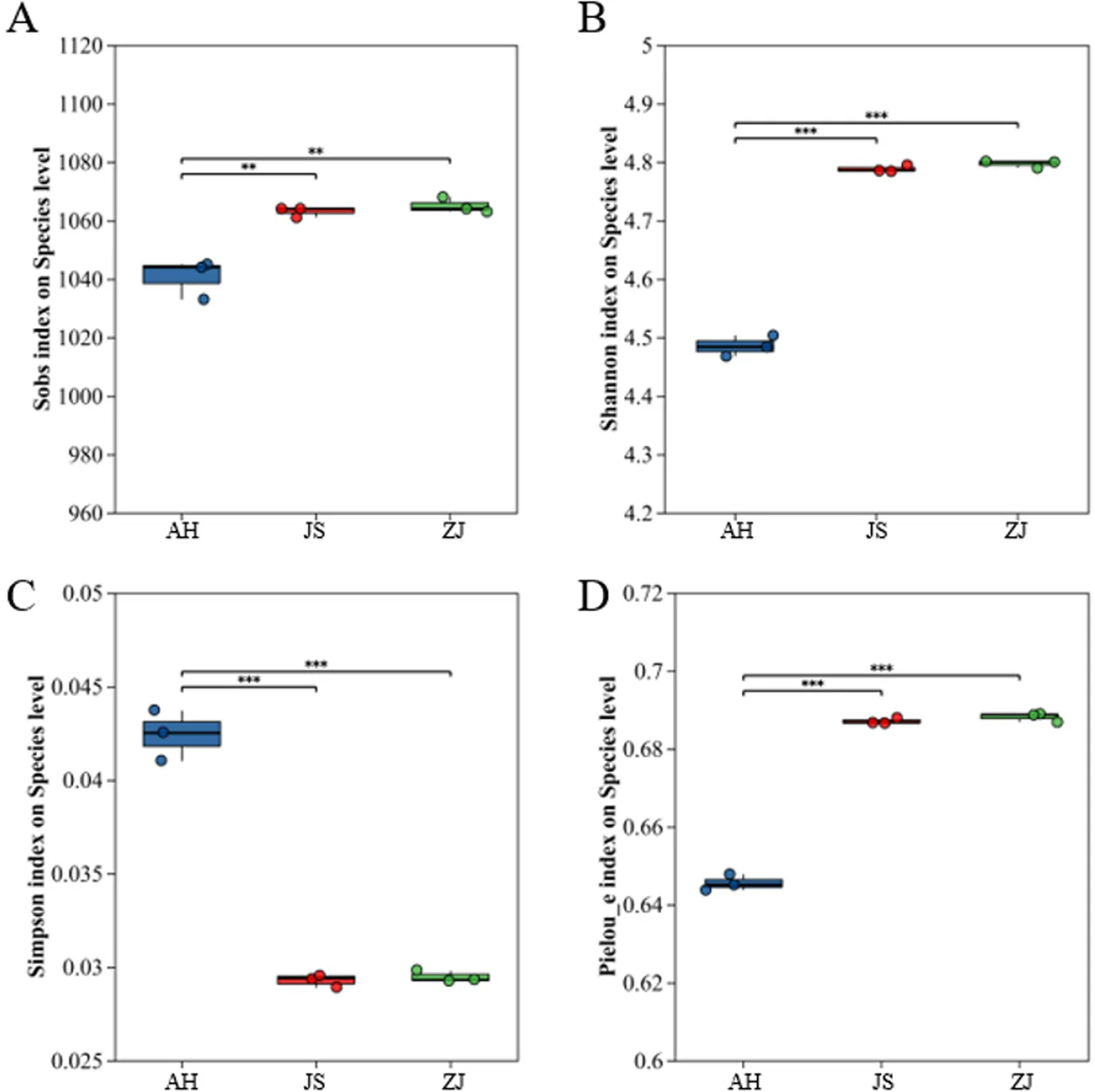
Comparison of bacterial α-diversity indices in traditional fermented amaranth stems collected from Anhui (AH), Jiangsu (JS), and Zhejiang (ZJ), China. **(A)** Sobs index; **(B)** Shannon index; **(C)** Simpson index; and **(D)** Pielou evenness index. Dots represent individual replicates, and horizontal lines indicate the mean values. Statistical significance between groups is indicated as follows: ** *p* < 0.01 and *** *p* < 0.001.

### 3.5 Bacterial community composition of traditional fermented amaranth stems

After removal of chloroplast- and mitochondrial-derived sequences, bacterial community composition was recalculated and renormalized to better represent the bacterial microbiota of fermented amaranth stems. At the phylum level, Firmicutes and Proteobacteria dominated all samples (Figure 4A). Firmicutes accounted for approximately 77%, 75%, and 56% in AH, JS, and ZJ, respectively, whereas Proteobacteria represented approximately 23%, 25%, and 40%; a small proportion of Bacteroidota was observed in ZJ. At the genus level, Lactobacillus, Lactococcus, and Pseudomonas were the major genera (Figure 4C). AH showed a higher abundance of Lactobacillus (approximately 52%), whereas Lactococcus was more abundant in JS and ZJ (approximately 42 and 41%). Pseudomonas accounted for approximately 23–25% across the three groups, while ZJ additionally contained higher proportions of Comamonas, Chryseobacterium, Massilia, Flavobacterium, Delftia, and Rheinheimera. PCA confirmed clear separation among the three groups: PC1 and PC2 explained 79.21 and 18.04% of the phylum-level variance and 64.87 and 16.12% of the genus-level variance, respectively (Figures 4B,D). These differences may be associated with multiple ecological and processing factors, including regional temperature and humidity, season, raw-material epiphytic microbiota, salt concentration, fermentation duration, water source, vessels, and local production practices (Yuan et al., 2024). However, these factors were not experimentally controlled in the present cross-sectional design; therefore, the observed differences are interpreted as region-associated patterns rather than direct climatic or geographical effects. Because the V4 amplicon did not provide sufficiently robust species-level resolution, species-level abundance and PCA results are not presented or discussed.

**Figure 4:**
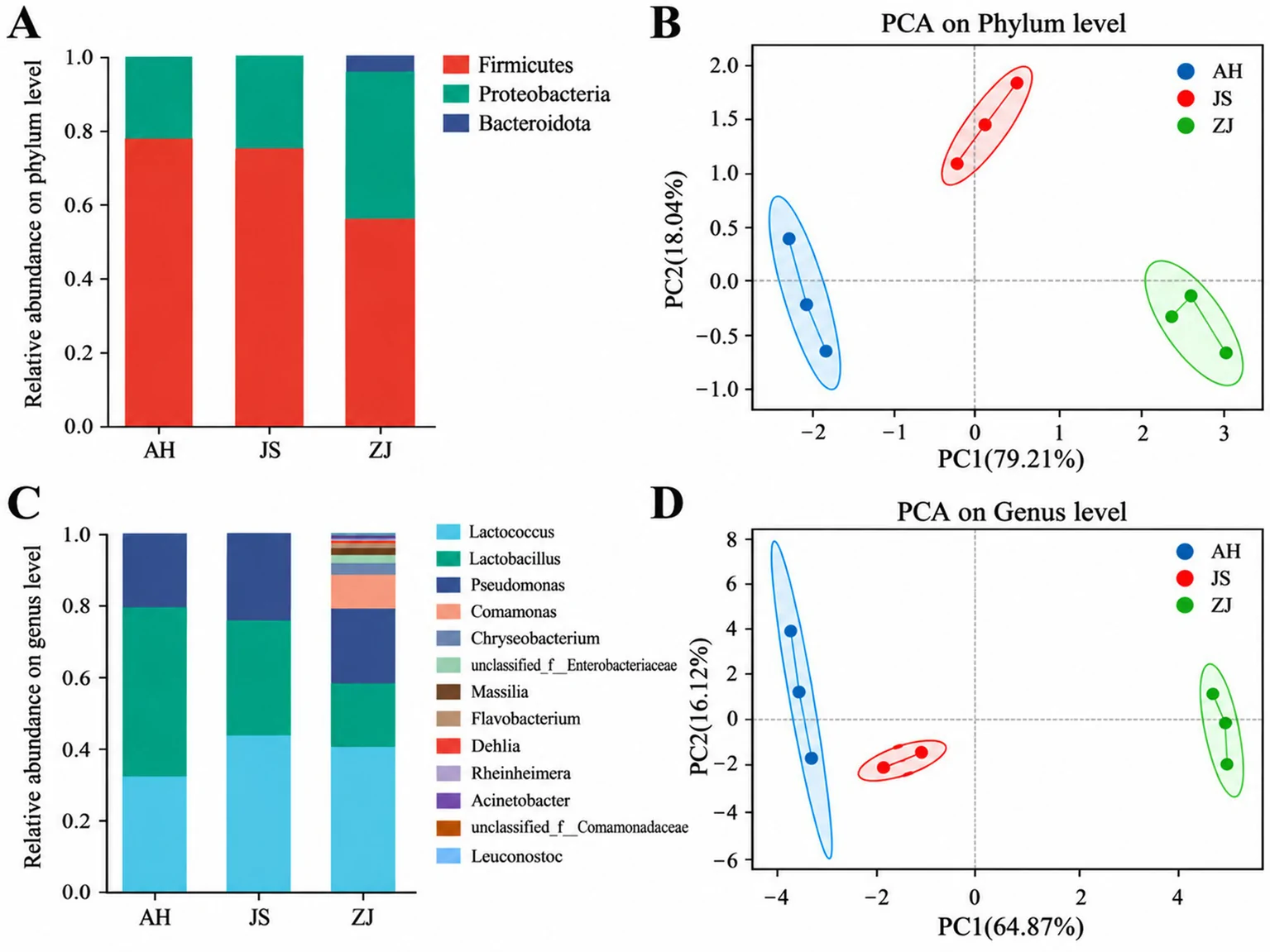
Bacterial community composition and principal component analysis (PCA) of traditional fermented amaranth stems after removal of chloroplast- and mitochondrial-derived sequences. **(A)** Relative abundance of major bacterial phyla; **(B)** PCA based on phylum-level composition; **(C)** Relative abundance of major bacterial genera; and **(D)** PCA based on genus-level composition. Relative abundances were recalculated and renormalized after organelle-derived sequences were excluded. Each point represents an independent sample. Species-level results are not shown because the V4 16S rRNA amplicon did not provide sufficiently robust species-level resolution.

### 3.6 Functional annotation of the metaproteome in fermented amaranth stems

Across the nine samples, 7,158 unique protein accession records were identified. Supplementary Table S1 provides the corresponding quantitative abundances, functional annotations, and taxonomic assignments. Of these proteins, 3,926 (54.85%) received taxonomic assignments, including 2,281 bacterial, 1,633 fungal, 10 archaeal, and 2 viral proteins; no taxonomically assigned entries were classified as plant-derived proteins. The remaining 3,232 proteins lacked confident taxonomic assignments and were therefore not attributed to specific microorganisms in taxon-specific interpretations.

Although dedicated fungal community profiling by ITS sequencing was not performed, the metaproteomic taxonomic annotation detected 1,633 proteins assigned to fungi. This finding suggests that fungal activity may contribute to the fermentation ecosystem; however, protein-level taxonomic assignments cannot substitute for a systematic fungal-community survey. We therefore refrain from drawing conclusions about fungal community composition or bacteria–fungi interactions and explicitly identify this as a limitation that should be addressed by ITS amplicon sequencing or shotgun metagenomics in future studies.

Functional annotation revealed that the proteins identified from fermented amaranth stems were predominantly associated with fundamental metabolic and cellular processes (Figure 5). In the GO classification, cellular process and metabolic process were the dominant biological-process categories, while cell/cell part represented the major cellular-component annotation (Figure 5A). At the molecular-function level, catalytic activity and binding accounted for the largest fractions, indicating extensive enzyme-mediated biochemical transformation during fermentation. Such a functional pattern is consistent with metaproteomic studies showing that fermented-food microbiomes express abundant enzymes involved in central metabolism, protein synthesis and environmental adaptation (Peng et al., 2025).

**Figure 5:**
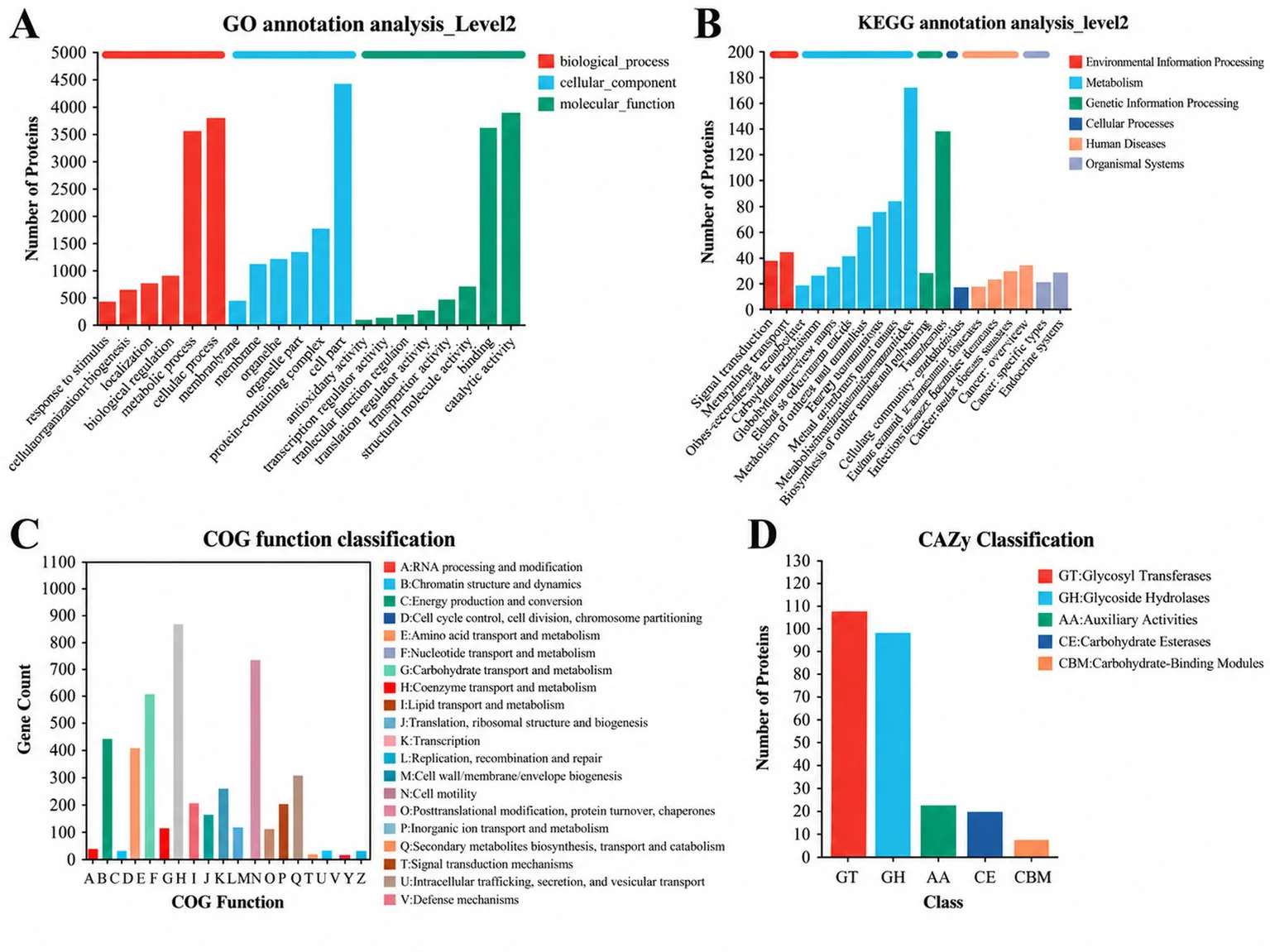
Functional annotation and classification of proteins identified from traditional fermented amaranth stem samples collected from three regions. **(A)** Gene Ontology (GO) functional classification at level 2, including biological process, cellular component, and molecular function categories. **(B)** Kyoto Encyclopedia of Genes and Genomes (KEGG) pathway annotation at level 2, including environmental information processing, metabolism, genetic information processing, cellular processes, human diseases, and organismal systems. **(C)** Cluster of Orthologous Groups (COG) functional classification of the identified proteins. Letters A–Z represent the corresponding COG functional categories indicated in the legend. **(D)** Carbohydrate-Active enzymes (CAZy) classification of proteins involved in carbohydrate metabolism, including glycosyl transferases (GTs), glycoside hydrolases (GHs), auxiliary activities (AAs), carbohydrate esterases (CEs), and carbohydrate-binding modules (CBMs). Bar heights represent the numbers of proteins assigned to each functional category.

KEGG annotation further demonstrated the predominance of metabolism, particularly global metabolic pathways, carbohydrate metabolism, amino acid metabolism and energy metabolism (Figure 5B). Translation-related proteins were also highly represented within genetic information processing. This pattern was supported by the COG classification, in which translation, ribosomal structure and biogenesis (J) was the most abundant functional category, followed by post-translational modification, protein turnover and chaperones (O), carbohydrate transport and metabolism (G), energy production and conversion (C), and amino acid transport and metabolism (E) (Figure 5C). The enrichment of these functions suggests an actively adapting fermentation ecosystem in which microorganisms require efficient protein synthesis, stress-response machinery and carbon/nitrogen utilization. Similar enrichment of glycolytic enzymes, translation machinery, chaperones and carbohydrate-processing proteins has been reported in metaproteomic analyses of fermented foods (Bahule et al., 2024).

CAZy annotation provided further evidence for intensive carbohydrate transformation (Figure 5D). Glycosyl transferases (GT; 107 proteins) and glycoside hydrolases (GH; 99 proteins) were markedly more abundant than auxiliary activities (AA; 22), carbohydrate esterases (CE; 19) and carbohydrate-binding modules (CBM; 7). GHs potentially facilitate hydrolysis of plant-derived polysaccharides and oligosaccharides, whereas GTs participate in glycan biosynthesis and remodeling. Together with the enrichment of carbohydrate and amino-acid metabolism, these results indicate that fermentation of amaranth stems involves extensive restructuring of plant carbohydrates and nitrogenous substrates, potentially supplying precursors for organic acids and characteristic volatile metabolites (Okeke et al., 2021). Metaproteomics is therefore particularly valuable for linking microbial composition with the actually expressed functional machinery of fermented foods (de Paula de Sousa Cavalcante et al., 2025).

### 3.7 Regional differences in functional protein profiles of fermented amaranth stems

The functional profiles of fermented amaranth stems differed among AH, JS, and ZJ, indicating region-associated variation in expressed functions (Figure 6). GO analysis showed that JS was relatively enriched in proteins associated with metabolic process, localization, cellular process, catalytic activity, and binding, whereas ZJ showed comparatively strong response-to-stimulus and biological-regulation signals (Figure 6A). Developmental-process annotations were interpreted cautiously because GO terms alone do not establish taxonomic origin. Taxon-specific conclusions were restricted to proteins with microbial assignments in Supplementary Table S1, avoiding attribution of unassigned proteins to microorganisms.

**Figure 6:**
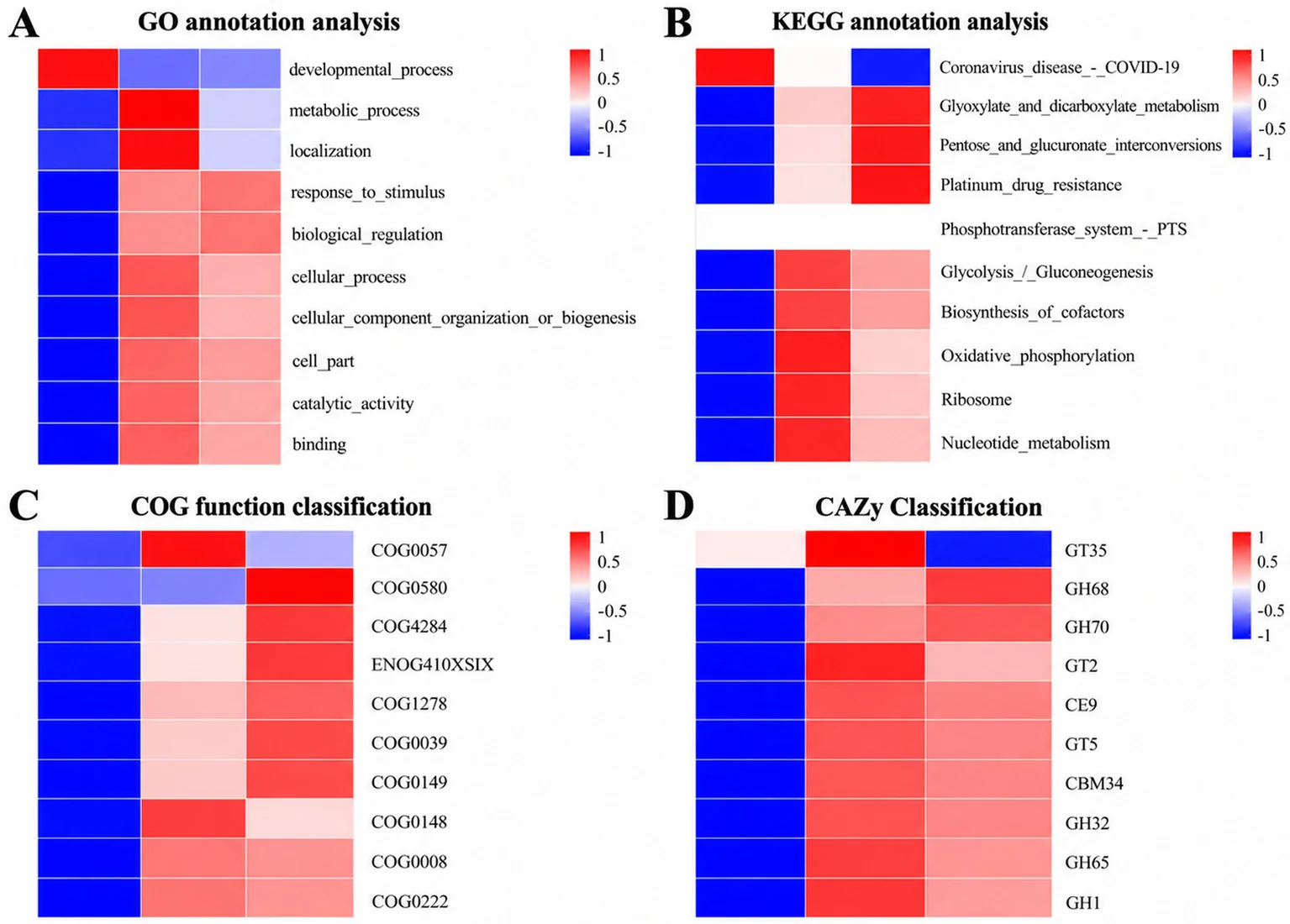
Comparative functional annotation of proteins identified in traditional fermented amaranth stems. Heatmaps show representative differential functional categories based on **(A)** GO, **(B)** KEGG, **(C)** COG, and **(D)** CAZy. Colors represent row-wise standardized protein abundance (*Z*-scores), with red indicating relatively higher abundance and blue indicating relatively lower abundance within each functional category.

KEGG analysis further demonstrated the metabolic differentiation. JS showed higher relative abundance of proteins involved in glycolysis/gluconeogenesis, cofactor biosynthesis, oxidative phosphorylation, ribosome, and nucleotide metabolism, collectively (Figure 6B). In contrast, the protein profile of ZJ was strongly enriched in glyoxylate and dicarboxylate metabolism and pentose and glucuronate interconversions, suggesting enhanced utilization of plant-derived carbohydrates and organic-acid generation. This is consistent with previous work showing intensive carbohydrate conversion during amaranth-stem fermentation and the important contribution of microbial metabolism to flavor formation (Peng et al., 2025). In COG profiles, ZJ were enriched in COG0580, corresponding to a glycerol uptake facilitator involved in carbohydrate transport and metabolism. While COG1278, a cold-shock protein associated with environmental adaptation was rich in ZJ sample (Figure 6C). Thus, ZJ may possess stronger substrate-transport and stress-response capacity.

The differential carbohydrate processing was confirmed by the CAZy annotation (Figure 6D). JS was relatively enriched in GT35, GT2, GT5, GH32, GH65, and CBM34, indicating extensive glycan synthesis, hydrolysis, and carbohydrate binding. In contrast, ZJ showed significantly enrichment of GH68 and GH70. Characterized GH68 enzymes include levansucrases and inulosucrases, whereas GH70 contains glucansucrases and glucan-modifying enzymes, implying the higher level of sucrose-dependent or glucan transformation in ZJ (Yakovlieva and Walvoort, 2020).

### 3.8 Integrated 16S rRNA–metaproteomic analysis reveals potential microbial–functional interactions during fermented amaranth stem fermentation

The correlation networks showed associations between dominant bacterial genera and expressed functional categories (Figure 7). Because correlations were calculated across nine individual biological samples, the networks are interpreted as exploratory microbial–functional associations rather than evidence of causation. In the GO network, Lactococcus, Lactobacillus, Leuconostoc, Pseudomonas, Flavobacterium, and Massilia were associated with catalytic activity, binding, localization, metabolic process, response to stimulus, biological regulation, and detoxification, suggesting links between community composition, substrate conversion, and environmental adaptation.

**Figure 7:**
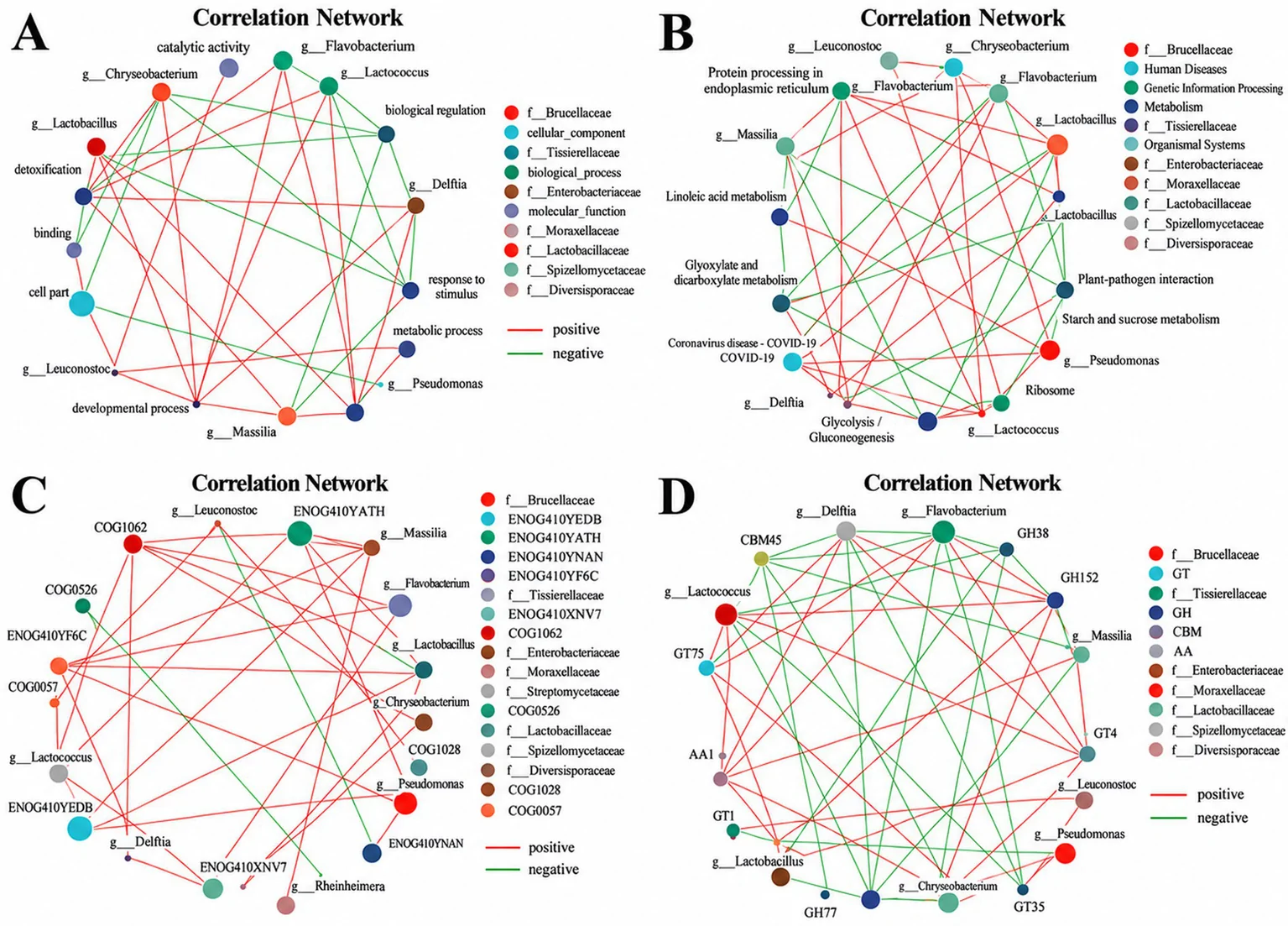
Integrated correlation networks linking dominant bacterial genera identified by 16S rRNA sequencing with metaproteomic functional annotations in traditional fermented amaranth stems. **(A)** GO categories; **(B)** KEGG pathways; **(C)** COG functions; and **(D)** CAZy families. Correlations were calculated across nine individual biological samples. Only exploratory associations with |*r*| > 0.6 and *p* < 0.05 are shown. Node size is proportional to relative abundance. Red and green edges indicate positive and negative correlations, respectively, and edge thickness reflects correlation strength.

The KEGG network further clarified these ecological roles. Lactic acid bacteria-related genera, especially *Lactococcus*, *Lactobacillus*, and *Leuconostoc*, were closely associated with glycolysis/gluconeogenesis, ribosome, and starch and sucrose metabolism, indicating their central role in sugar fermentation, protein synthesis, and likely organic acid production (Figure 7B). In contrast, *Pseudomonas*, *Flavobacterium*, and *Massilia* showed connections with glyoxylate and dicarboxylate metabolism and protein processing that have suggested broader ecological functions in plant tissue degradation, metabolite turnover, and environmental adaptation. The coexistence of positive and negative correlations indicates both cooperation and competition among taxa, which is typical of spontaneous vegetable fermentation (Sun et al., 2023; Xu et al., 2021).

The COG and CAZy networks provided additional mechanistic context. Associations with GH, GT, CBM, and AA families are consistent with carbohydrate deconstruction, glycan transformation, and microbial adaptation (Figures 7C,D). When considered together with the measured flavor precursors, plausible pathways include microbial utilization of tryptophan toward indole-related metabolism, phenylalanine conversion toward aromatic alcohols, and carbohydrate hydrolysis followed by central carbon metabolism that supplies organic-acid and ester precursors. These pathway links remain hypotheses derived from cross-sectional multi-omics associations and require targeted enzyme assays, metabolite tracing, or controlled inoculation experiments for causal validation.

### 3.9 Study limitations and future perspectives

Several limitations should be considered when interpreting the present results. First, this was a cross-sectional study involving only three production regions with three samples per region, and processing parameters such as fermentation time, temperature, salt concentration, and local environmental conditions were not experimentally standardized. Second, 16S rRNA sequencing characterized only the bacterial component, while fungal community composition and bacteria–fungi interactions were not directly resolved. Third, the microbiome–metaproteome networks are correlation-based and cannot establish causal relationships between specific taxa, proteins, and flavor formation. Finally, targeted enzyme assays, isotope/metabolite tracing, and controlled inoculation experiments were not performed. Future studies should therefore include additional regions, seasons, and independent production batches; record or standardize processing parameters; integrate bacterial and fungal community profiling; and validate candidate microbial–functional links using controlled fermentation, targeted metabolomics, enzyme assays, and strain-level inoculation experiments.

## 4 Conclusion

This study demonstrates that traditional fermented amaranth stems from three production regions differ not only in bacterial community composition but also in expressed metaproteomic functions and flavor-related traits. The combined 16S rRNA–metaproteomic framework highlights plausible links between dominant bacteria, carbohydrate-active enzymes, central metabolism, and flavor-precursor transformation. These associations should be viewed as mechanistic hypotheses rather than causal proof. By defining the functional features that warrant targeted validation and by identifying key sources of variation that require better process control, this work provides a basis for future controlled studies aimed at understanding and improving the quality consistency of fermented amaranth stems.

## Data Availability

The datasets presented in this study can be found in online repositories. The names of repository and accession number(s) can be found in the article/Supplementary material.
